# Evaluation of a Novel Teleradiology Technology for Image-Based Distant Consultations: Applications in Neurosurgery

**DOI:** 10.3390/diagnostics11081413

**Published:** 2021-08-04

**Authors:** Paulina Cewe, Gustav Burström, Ivan Drnasin, Marcus Ohlsson, Halldor Skulason, Stanislav Vucica, Adrian Elmi-Terander, Erik Edström

**Affiliations:** 1 Department of Trauma and Musculoskeletal Radiology, Karolinska University Hospital, 171 64 Stockholm, Sweden; paulina.cewe@ki.se; 2 Department of Clinical Neuroscience, Karolinska Institutet, 171 77 Stockholm, Sweden; marcus.ohlsson@sll.se (M.O.); adrian.elmi-terander@sll.se (A.E.-T.); erik.edstrom@sll.se (E.E.); 3 Department of Neurosurgery, Karolinska University Hospital, 171 64 Stockholm, Sweden; 4 Image Over Globe, 21000 Split, Croatia; ivan.drnasin@ioverglobe.com (I.D.); svucica@metecons.com (S.V.); 5 Department of Neuroradiology, Karolinska University Hospital, 171 64 Stockholm, Sweden; 6 Department of Neurosurgery, Landspitali University Hospital, 101 Reykjavik, Iceland; halldor.skulason@me.com

**Keywords:** remote consultation, teleradiology, clinical decision-making, telemedicine, neurosurgery

## Abstract

In emergency settings, fast access to medical imaging for diagnostic is pivotal for clinical decision making. Hence, a need has emerged for solutions that allow rapid access to images on small mobile devices (SMD) without local data storage. Our objective was to evaluate access times to full quality anonymized DICOM datasets, comparing standard access through an authorized hospital computer (AHC) to a zero-footprint teleradiology technology (ZTT) used on a personal computer (PC) or SMD using national and international networks at a regional neurosurgical center. Image datasets were sent to a senior neurosurgeon, outside the hospital network using either an AHC and a VPN connection or a ZTT (Image Over Globe (IOG)), on a PC or an SMD. Time to access DICOM images was measured using both solutions. The mean time using AHC and VPN was 250 ± 10 s (median 249 s (233–274)) while the same procedure using IOG took 50 ± 8 s (median 49 s (42–60)) on a PC and 47 ± 20 s (median 39 (33–88)) on a SMD. Similarly, an international consultation was performed requiring 23 ± 5 s (median 21 (16–33)) and 27 ± 1 s (median 27 (25–29)) for PC and SMD respectively. IOG is a secure, rapid and easy to use telemedicine technology facilitating efficient clinical decision making and remote consultations.

## 1. Introduction

Digitalization of medical imaging in combination with rapid evolution of personal computers (PC) and small mobile devices (SMD), has unlocked new possibilities to availability, communication and diagnostic evaluation of medical imaging [1]. Recent advances in teleradiology have facilitated the emergence of several software technologies for accessing radiological examinations on mobile devices [2]. In emergency settings, such as head trauma or stroke, fast access to medical imaging for diagnostic evaluation is pivotal for clinical decision making and optimized patient outcomes [3].

For some patients, external consultations are necessary requiring images transfer outside the secure hospital networks. Image transfer based on teleradiology provides diagnostic services to patients anywhere in the world and technologies utilizing SMD, may prove useful not only in rural areas of developing countries but also in parts of the world where infrastructure is lacking. Hence, on 7 June 2011 the World Health Organization (WHO) issued a statement regarding the need for technical solutions based on small and low-cost, handheld, devices such as tablets and mobile phones [4].

Within modern hospitals image datasets are stored in a digital archive system, called picture archiving and communication system (PACS). An international digital imaging standard for radiographic images was established in the late 1980s, to harmonize communication, storage and archiving of medical image information; namely, digital imaging and communications in medicine (DICOM). Initially a radiology-specific standard, it has since then expanded to include many other areas in medicine [5]. The DICOM standard is used in all Swedish hospitals since medical imaging was digitized in the mid-1990s [6].

Digitalization, internet development and improved networking solutions have established methods of transferring radiological images between hospitals [7]. International referrals are facilitated via secure transfer of images [8]. However, the process involves several time consuming manually operated steps, potentially having a negative impact on rapid diagnostic evaluation and patient outcomes [3]. Transferring images outside a secure health care network is even more challenging as it requires an approved computer, additional user accounts with remote access and a virtual private network (VPN) connection.

In 2016, a zero-footprint teleradiology technology named Image Over Globe (IOG), CE marked with Class IIb classification, was introduced clinically at the study center. The hospital is a Level 1 trauma center and the Neurosurgical Department is the primary referral center for approximately 2.5 million inhabitants. In this study, we evaluate this teleradiology solution, aimed at solving the problem of secure and efficient remote consultations using PC or SMD outside the hospital network.

In neurosurgical practice, numerous consultations and evaluations of medical images are performed every day [9,10,11]. Access to high quality images is essential for clinical decision making. Remote consultations are often difficult, as the transfer of images between regions or countries must be performed outside the secure health care networks. Another situation requiring data transfer outside the secured networks is during off-hours. At these hours, consultations are mainly limited to emergency cases in need of immediate attention. A junior neurosurgeon is often on-call at the hospital while senior colleagues are on-call at home.

Moreover, as of 25 May 2018 the General Data Protection Regulation (GDPR), a regulation concerning the protection of confidentiality of personal data, was implemented by the European Union (EU). Among the many areas regulated by GDPR, are data access for patients, data processing and explicit patient consent in the absence of exemptions. It also regulates matters such as encryption, anonymization and pseudonymization [12]. In addition to GDPR, Swedish legislation includes “The Public Access to Information and Secrecy Act”, “The Patient Data Act” and “The Patient Safety Act” [13]. As defined by these regulations, patient datasets cannot simply be up- and down-loaded to and from public web pages, nor can they be sent by email.

**Figure 1:**
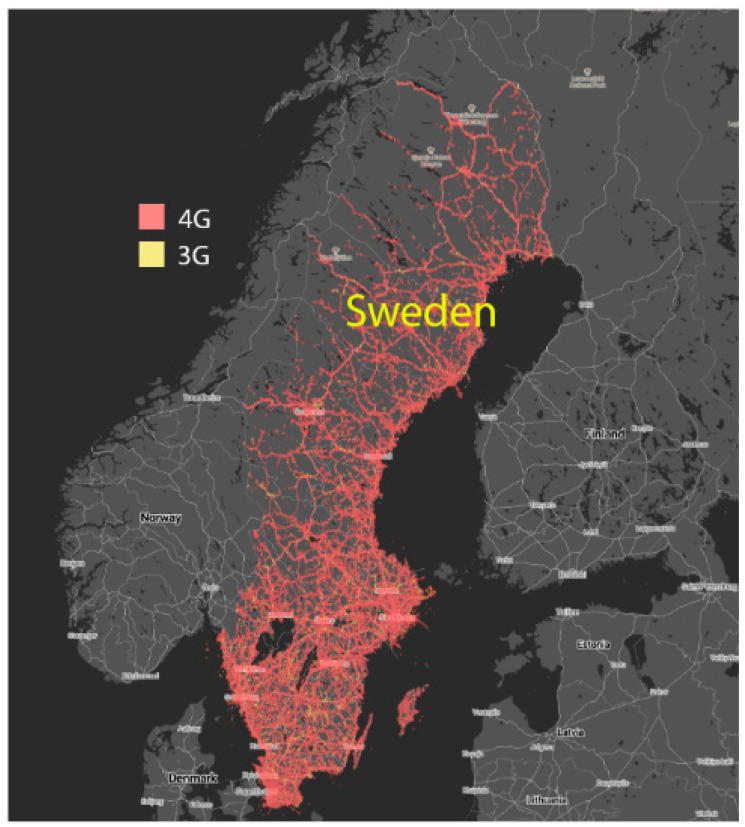
Sweden is a country with a dense grid of mobile phone masts. The map illustrates 4G (red) and 3G (yellow) mobile technology coverage in Sweden provided by Telia. Image reproduced with permission from www.tutela.com (accessed on 25 September 2020).

Sweden has an extensive mobile 3G and 4G network coverage, encompassing 99% of the country’s inhabitants (Figure 1). The teleradiology technology in this study is dependent on the mobile infrastructure and consequently, testing in areas where network coverage is unsatisfactory would only prove this fact. Instead, comparison of direct time-to-access was made between an authorized hospital computer (AHC) and IOG using either PC or SMD. In addition, evaluation of IOG in long distance consultations with the Neurosurgical Center at Landspital in Reykjavik, Iceland was performed. An agreement to collaborate internationally on certain neurosurgical cases has existed for many years. However, no infrastructure for rapid access to patient imaging data exists.

## 2. Materials and Methods

### 2.1. Basic Characteristics of the Teleradiology Technology

IOG (Infomedica, Split, Croatia) is a CE-marked product designed to support global medical image access and clinical collaboration. It is a cross-platform technology, utilizing web access to reach any desktop, tablet or mobile device through a standard web browser. IOG is built around a microservice architecture with a cloud segment and on-premise segment:

- The cloud segment is installed in AWS’s (Amazon Web Services, Seattle, Washington, USA) HIPAA (Health Insurance Portability and Accountability Act) compliant data center and manages all non-image-related services, like authentication, authorization, security, sharing, integration, APIs, and hosting.
- The on-premise segment is installed locally in the hospital. The main purpose is to authorize user image access to PACS and to send DICOM images directly (no cloud involved) to the user’s web browser, without storing images outside of the hospital.

As such, IOG enables secured web-based full diagnostic image quality access, bearing CE Class IIb medical product mark.

By utilizing IOG integration services, IOG is integrated with Swedish BankID secured communication platform and provides image dataset distribution options to other IT systems such as Neuro:Q (Nucleoniq, Eindhoven, The Netherlands) to be employed in emergency end-to-end careflow applications, e.g., stroke or TBI.

### 2.2. Installation and Requirements of the Teleradiology Technology

**Figure 2:**
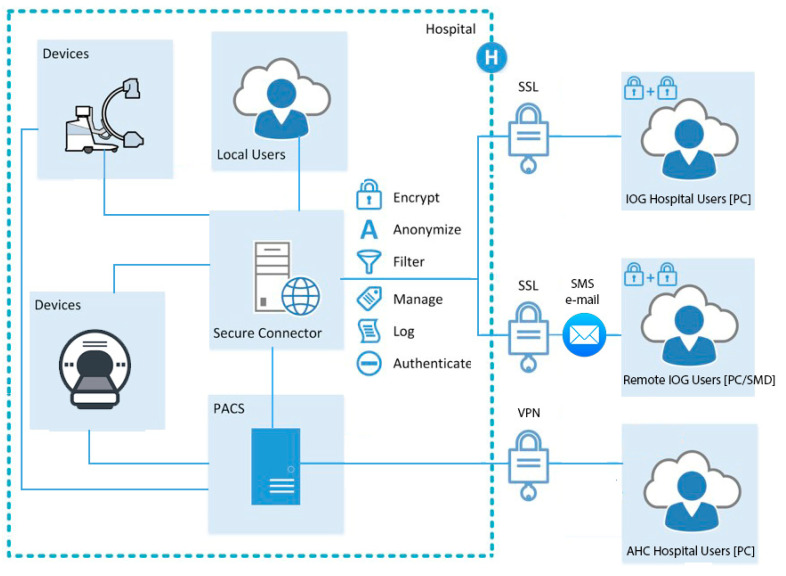
Principles for AHC and IOG accessing PACS from outside the hospital network while staying compliant with applicable patient data regulations. The AHC uses a predefined user account and a VPN solution to enable access to the secure hospital network while IOG uses two step authentication and a preinstalled connector which acts as a relay between the user and the hospital network. This connector also enables invited users to view anonymized image data via a link provided by a user.

The IOG platform requires a web browser and internet connection. Patient datasets, stored in PACS, are accessed using standard DICOM protocol via a dedicated connector, which communicates “on-demand”, and uses secure web access communication. The connector is installed in a secured area of the hospital network. It is isolated from the hospital network and utilizes both DICOM and HTTPS protocols to communicate with PACS and mobile web clients (Figure 2). The connector retrieves datasets and provides them on a PC or SMD with suitable display capabilities, inside or outside the hospital network. The choice of mobile technology (mobile phone, tablet, or laptop) is arbitrary, as the technology uses a web browser and does not require any software installation. Once communication is established, the teleradiology application serves as a portal to the PACS server, through the connector (Figure 2). Images are transferred through reversible lossless GZIP compression to ensure full diagnostic image quality [14].

### 2.3. Standard Workflows to Access DICOM Datasets

Consultant neurosurgeons are provided with an AHC, by the hospital IT unit to remotely connect to PACS when on-call at home. Connection is established via VPN. Alternatively, the teleradiology technology IOG is used. A secure connection is established with a two-step authorization protocol. AHC and IOG workflows are presented in Table 1.

### 2.4. Hardware for Speed Tests of Standard and Teleradiology Technologies

**Figure 3:**
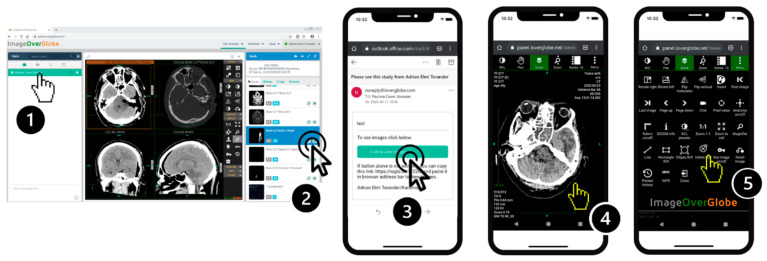
Workflow for remote consultations: Step 1. User selects predefined remote consultant in the application. Step 2. User selects series to be sent anonymized to the consultant through e-mail. Step 3. Consultant receives an e-mail with a URL to the complete set of anonymized series. Step 4. Images are instantly accessed on either SMD or PC. Step 5. Several tools such as measurements, line drawing, MPR-function, are available to facilitate clinical interpretation of lossless images.

The baseline workflow evaluations, presented in Table 2 and visualized in Figure 3, were performed using a PC (DELL desktop, Intel Xeon, 3.5 GHz, Windows 7 enterprise, 16 GB RAM, Google Chrome) and an SMD (iPhone X, iOS 11, Safari). The speedtest.net web application was used to test and establish reference internet speed.

In clinical evaluations, an AHC (Dell Latitude 5580, Intel Core i54-6440HQ, 2.6 GHz, Windows 7, 16 GB RAM), was used to connect to hospital PACS through standard secure access protocols (VPN). It was compared with IOG access to the PACS run on either a standard computer (HP Omni 27-1209eo, Intel Core i5-3330s, 2.70 GHz, Windows 10, 16 GB RAM) or standard mobile phone (Samsung Galaxy S9) using IOG.

The display size of the SMDs ranged from 4.7 to 9.7 inches. The high spatial resolution, acceptable contrast ratios and brightness levels, provided good image quality for safe interpretation [15,16]. DICOM images were displayed in original quality without any pixel loss and image quality remained intact while zooming. The teleradiology application provided touch screen gestures for image stacking, modifying window-width and level, as well as zooming/panning.

### 2.5. Teleradiology Technology Compliance to GDPR and Swedish Regulations

To stay fully compliant with GDPR and national regulations, an extended two-step authentication was used whereby predefined users were allowed to login using a user-defined username and password. Subsequently, a software generated SMS code, utilizing a continuously changing unique identifier, was sent to the user’s predefined phone number to be resubmitted for authentication. The patient data and images are safely transmitted from sender to receiver by means of an encrypted transport layer security (TLS) protocol between the connector and web browser. PACS sends non-anonymized data to the connector, and anonymization is performed automatically before data is transmitted outside the hospital network. Thus, patient data is anonymized for all links sent to non-IOG users (Table 1, Figure 2). The DICOM viewer uses a zero-footprint technology to ensure that no patient data are stored on accessing devices.

### 2.6. Workflow Efficacy of the IOG Teleradiology Technology for Standardized Datasets

To evaluate baseline workflow efficacy of IOG, three standard image datasets of variable sizes were selected from the publicly accessible Brainix MRI study (DICOM image sample sets, Osirix DICOM Image Library). Measurements were performed in two separate sessions, to observe possible transfer rate variations (Table 2). The first series (sT2W-FLAIR) was used in the first session, while the other two series were used in the second session (T1-SE-extra and T1-3D-FFE-C-801). For each image set, measurements were repeated 10 times.

### 2.7. Comparison of Access Times for AHC and IOG to Clinical DICOM Datasets

To compare access times to clinical DICOM datasets, for AHC and IOG on a PC or SMD, a series of tests were performed where a junior doctor consulted a senior doctor on-call at home using either of the two systems. All tests were performed with the senior doctor using a regular home network connection (Telia broadband 10/100 Mbps up/download, 1 Gbps WiFi (ASUS RT-AC3200)), and the junior doctor using regular PC on a broadband connection.

For AHC access, measurements began from starting the computer to connecting to the secure VPN, and from accessing PACS to receiving the complete image-set using a search query with patient ID.

For IOG access, measurements were made separately for PC and SMD. In the PC test, a call was simulated in the same fashion as for testing AHC access time. The senior doctor accessed the IOG platform on a PC kept in sleep mode at the start of the test. Access required two-step authentication using both user credentials and SMS to a registered mobile phone. Patient IDs were used to search for image-sets. Time was measured from answering the initial call to receiving the complete image-set. In the second test, a similar call was simulated and the senior doctor used a SMD to access the image-set, also requiring two-step authentication (Figure 2 and Figure 3, Table 3). For each image set, measurements were repeated 10 times.

### 2.8. Accessing Patient DICOM Datasets Using IOG on PC or SMD Per Invitation

The third test studied the IOG functionality of facilitating transfer of anonymized image-sets over a secure connection. In this test, the junior doctor used IOG on a PC (requiring two-step authentication) to access the image-sets which were then made available to the senior doctor through a secure link, sent as SMS or email. When clicked, the link leads to an anonymized version of the image-set. Time was measured from the junior doctor initiating IOG to the senior doctor receiving the full image-set on an SMD (Figure 3, Table 3).

### 2.9. International Consultation Using IOG on PC or SMD

The final test was an international consultation between neurosurgeons at two different hospitals. An invitation to access a patient DICOM data was sent from Iceland to Sweden. The invitation was sent per email or SMS. On a simultaneous phone call, time from sending the email access link until the full image dataset was accessed was measured (Table 3). No comparison was made to the standard access method since the only other way of transferring image datasets between Iceland and Sweden is through international postal services.

### 2.10. Statistical Analysis

Time was expressed as means ± standard deviations and as medians [interquartile ranges]. A two-sided Student’s t-test was used for comparing means between groups. Statistical analysis was performed using RStudio (RStudio Team (2016). RStudio: Integrated Development for R. RStudio, Inc., Boston, MA, USA) and a *p* value less than 0.05 was considered as significant. All data points represent 5 repeated measurements and results are presented in Table 2 and Table 3.

## 3. Results

### 3.1. Subsection Workflow Efficacy Measurements of the IOG Teleradiology Technology

Transfer times and time to perform searches were recorded for each step of the generic workflows (described in Table 1). A sub-analysis was performed on image transfer measurements, since transfer duration was a factor adding to the total time to receive images remotely. The measured compression levels, download speeds and corresponding transfer times for standard image-sets, indicate stable lossless compression levels of 34–38% and median transfer times of 2–5 s (Table 2).

### 3.2. Clinical Efficacy Measurements of DICOM Dataset Access Using AHC or IOG

The results for simulated distant consultations using AHC or IOG are presented in Table 3. The mean time using AHC was 250 ± 10 s (median 249 s (233–274)) while the same procedure via IOG took 50 ± 8 s (median 49 s (42–60)) on a PC and 47 ± 20 s (median 39 (33–88)) on an SMD. Using IOG resulted in a significant reduction (80%) of the time needed compared to accessing DICOM dataset using VPN from home (50 ± 8 s versus 250 ± 10 s; Table 3).

### 3.3. Accessing Patient DICOM Datasets Using IOG on PC or SMD Per Invitation

Simulating a consultation initiated by an IOG user sending a link by SMS took less than 70 s (Table 3). This included both the times for the primary user and the invited user but was still significantly less than the time needed when using AHC to access DICOM datasets.

### 3.4. International Consultation Using IOG on PC or SMD

Results of the international consultation between Iceland and Sweden are similar to the corresponding times nationally, requiring roughly 30 s for image transfer regardless of device (Table 3).

## 4. Discussion

Complete medical image datasets are not easily shared outside secure hospital networks due to large file sizes and security restrictions regulated by national laws and GDPR. Even then, image transfer process involves several manually operated steps which may be time consuming and delay rapid diagnostic evaluation [3]. Transferring images outside a secure health care network is even more challenging as this requires an approved computer, additional user accounts with remote access and a VPN connection. In many cases, sending material on CDs or memory sticks by regular mail may be the only solution for second opinions. This may transfer data viruses into hospital networks. Teleradiology has been introduced globally during the latest decades. Modern medicine has evolved in synergy with technical advances in imaging, storage and networking unlocking new possibilities for communication, diagnosis and treatment [1]. However, with new technological leaps and easy access to personal data, rules and regulations had to be established to protect individual privacy. Hence, there is a need for a fast and secure solution for transferring radiological datasets between medical centers without violating regulations [17].

In this study, the use of the teleradiology technology IOG, at a neurosurgery department was described. The technology was introduced to bypass cumbersome administrative practices and outdated technical solutions for remote access by introducing a solution that runs on any PC or SMD [18].

There are previously published studies using similar software for mobile devices, but they have been retrospective and executed in a controlled environment [3]. One study measured time acquisition but used anonymization by converting DICOM images to JPEG which introduces a loss of image quality and prevents the ability of 2-D post processing [19]. Another study was conducted in a stroke referral center but focused on image quality and interrater agreement [20]. This study is the first to test fully anonymized and full diagnostic quality DICOM datasets in a regional neurosurgical center through national and international remote consultations.

The technology was tested on-call in parallel with available standard technology. As indicated by the results, time to access medical images was significantly lower when using IOG compared to AHC using VPN, regardless of method of access (Table 3, Figure 3). This is partly explained by the simplified workflow of IOG, where no time is spent starting the device or logging on to a VPN-service. A household PC or SMD is assumed to be continuously up and running (or in sleep mode). However, the AHC is typically switched off and securely stored. Time required for downloading datasets, and subsequently access times, was shortened by IOG, using the GZIP-format for reversible lossless compression. GZIP is an internationally established format for compression and preserves full diagnostic quality of images [14]. Compression reduced DICOM image size by more than 60%, resulting in transfer time reduction. Measured transfer times for SMD were less than 11 s compared to 25–30 s (data not shown) without compression, indicating that transfer times had a minor effect on total time for accessing image-sets even on slower mobile networks (Table 2). With larger file sizes due to increased image resolution, lossless image compression becomes increasingly important. Moreover, transfer time reduction is important when using mobile internet connections, often slower than hardwired connections.

IOG provides image access through SMS or email. This simplifies and shortens remote consultations, by removing query time. This is essential for remote consultations between different hospitals. International consultations have previously been dependent on image-sets being sent by regular mail. Similarly, remote consultations with Iceland have relied on international shipping. Using the current teleradiology technology, image-based consultations can now be performed within minutes. In a global perspective, an easily accessible teleradiology platform has the potential to improve accessibility to neurosurgical expertise in rural areas where it is lacking, and where the alternative may be long distance patient transferals [21,22].

Although not part of the systematic evaluation, IOG also provides functionality for online live audio and video conferencing, while sharing the same image datasets independently of physical locations and allows users to store selected image datasets anonymously in a private portfolio for future use.

In light of the recent COVID-19 pandemic, teleradiology provides a solution for image-based consultations between different parts of the world, simplifying the communication and discussion between consultants. There is also an opportunity to retain capacity, providing local neuroradiologists and neurosurgeons the possibility to work with consultations from home, thus limiting the need for outsourcing [23].

## 5. Limitations

The study was performed to illustrate the utility of a telemedicine solution. Comparisons of different speeds in part reflect the efficacy of the local technological infrastructure and available equipment and are expected to differ from results obtained in, e.g., another part of the world. Thus, the measurements will partly represent the available standard technology where and when the study was performed.

## 6. Conclusions

This modern teleradiology technology allows easy and rapid patient image access using mobile devices, in and outside the hospital. The communication between clinicians is significantly simplified. The patient privacy issue is adequately resolved by introducing the two safeguarding levels. The teleradiology technology Image over Globe is a safe, rapid and secure way of transferring DICOM data for rapid clinical decision making and distant consultations.

## Figures and Tables

**Table 1:** User query initiated DICOM access using either AHC or IOG.

	AHC User Initiated	IOG PC/SMD User Initiated	IOG PC/SMD Per Invitation
1	Requires predefined user status	Requires predefined user status	Receive SMS or email invitation link
2	Installed software login	Web application login	Using link bypasses login
3	Preinstalled VPN provides secure connection	Automatically generated code sent by email/smsCode returned to software for second step authorization	Link bypasses authorization
4	Query using patient name/ID	Query using patient name/ID	n/a
5	Accessing DICOM sets on PACS	Accessing DICOM sets on PACS through IOG connector	Accessing DICOM sets on PACS through IOG connector
6	Images viewed in application	Images viewed in application	Anonymized images viewed in application

Abbreviations: AHC—authorized hospital computer, DICOM—digital imaging and communications in medicine, IOG—Image Over Globe, PC—personal computer, SMD—small mobile devices.

**Table 2:** Baseline transfer times for standard image sets using lossless compression.

Series Name	No. of Images/Resolution	Total Original Series Size (MB)	Total Lossless Compressed Series Size (MB)	Total Lossless Compressed Series Size (% of Original Size)	PC Download Speed (Mbps)	SMD Download Speed (Mbps)	PC Median Transfer Time (s)	SMD Median Transfer Time (s)
“sT2W-FLAIR-401”	22/288 × 288	3.7	1.4	38%	88	59	2 (2–4)	2 (2–8)
“T1-SE-extra-701”	22/512 × 512	12.3	4.3	35%	78	16	4 (2–7)	3 (2–11)
“T1-3D-FFE-C-801”	100/256 × 256	14.2	4.9	34%	78	16	5 (5–6)	3 (3–8)

Abbreviations: MB—megabyte, Mbps—megabit per second, PC—personal computer, SMD—small mobile devices.

**Table 3:** Consultation times using IOG for distant access to clinical images.

Distant Consultations	Mean Time (s)	Median Time (s)	*p*-Value for Comparison to A.
**A. Regular hospital PACS using VPN on AHC**	250 ± 10	249 (233–274)	
Starting computer	127 ± 7	125 (122–135)	
Connecting to VPN	52 ± 9	49 (46–65)	
Opening PACS and images	71 ± 6	70 (65–77)	
**B. Senior on call using IOG on PC**	50 ± 8	49 (42–60)	*p* < 0.001
**C. Senior on call using IOG on SMD**	39 ± 5	38 (33–47)	*p* < 0.001
**D. Using IOG to send access link to senior on call**	68 ± 6	69 (59–82)	*p* < 0.001
Junior on call: starting IOG and opening images	36 ± 5	34 (33–42)	
Junior on call: sending access link by SMS to senior on call	19 ± 6	16 (15–25)	
Senior on call: image set opened using link on SMD	13 ± 2	12 (11–15)	
**E. Using IOG for international consultation Iceland-Sweden**			n/a
Providing access link from Iceland to Sweden by email (time from link sent until image viewed on PC)	23 ± 5	21 (16–33)	
Providing access link from Iceland to Sweden by SMS (time from access link sent until image viewed on SMD)	27 ± 1	27 (25–29)	

Abbreviations: AHC—authorized hospital computer, DICOM—digital imaging and communications in medicine, IOG—Image Over Globe, PACS—picture archiving and communication system, PC—personal computer, SMD—small mobile devices, VPN—virtual private network.

## Data Availability

Data is available from the corresponding author upon reasonable request.
